# Response to “Comment on ‘Association between Lifetime Exposure to Inorganic Arsenic in Drinking Water and Coronary Heart Disease in Colorado Residents’”

**DOI:** 10.1289/ehp.1509791R

**Published:** 2015-07-01

**Authors:** Katherine A. James, Tim Byers, John E. Hokanson, Jaymie R. Meliker, Julie A. Marshall

**Affiliations:** 1 University of Colorado Anschutz Medical Campus, Denver, Colorado, USA; 2 Stony Brook University, Stony Brook, New York, USA

We thank Rylander for his interest in our article and for suggesting magnesium may confound the association we observed between inorganic arsenic in water and risk of coronary heart disease. We did not include measures of drinking water magnesium (or hardness) in our study. However, levels of calcium and magnesium reported for the Rio Grande aquifer (which served our study population in the San Luis Valley) indicate higher levels of hardness, with low variability in the San Luis Valley (Robson and Banta 1995). In contrast, the arsenic level in groundwater in the San Luis Valley water quality samples showed considerable variability of 0–95 µg/L.

The correlation between magnesium and arsenic is unknown for the San Luis Valley aquifer. However, in response to Rylander’s letter, we calculated a Pearson correlation coefficient of 0.12 between arsenic and magnesium using arsenic data from Meliker et al. (2010) and unpublished magnesium data from a study in Michigan. We saw mean arsenic concentrations of 5.3, 0.6, and 6.5 µg/L for magnesium categories of < 4, 4–11, and > 11 mg/L, respectively, with a total sample size of 1,500 water samples and at least 400 samples in each category. These estimates do not support the hypothesis that arsenic and magnesium are highly correlated in drinking water, hence reducing concerns about confounding between these two minerals. More importantly, given that low levels of magnesium (Rylander 1996) and conversely high levels of arsenic (Moon et al. 2013) are thought to be associated with heart disease, any positive correlation between arsenic and magnesium would result in an underestimation (not overestimation) of the size of the association between inorganic arsenic exposure and coronary heart disease found in our study.

As with any observational epidemiologic study, it is important to continue to search for potential confounders and other risk factors. We continue to investigate the naturally occurring metals and minerals in the San Luis Valley drinking water supply and the potential associated long-term outcomes. This research will include investigating co-mixtures of elements and potential confounders that could be related to heart disease risk. We find Rylander’s letter thought provoking, and we thank him for bringing this evidence pertaining to magnesium to our attention.
